# Clonal Hematopoiesis of Indeterminate Potential and its Association with Treatment Outcomes and Adverse Events in Patients with Solid Tumors

**DOI:** 10.1158/2767-9764.CRC-24-0522

**Published:** 2025-01-09

**Authors:** Tharani Krishnan, Joao Paulo Solar Vasconcelos, Emma Titmuss, Robert J. Vanner, David F. Schaeffer, Aly Karsan, Howard Lim, Cheryl Ho, Sharlene Gill, Stephen Yip, Stephen K. Chia, Hagen F. Kennecke, Derek J. Jonker, Eric X. Chen, Daniel J. Renouf, Chris J. O’Callaghan, Jonathan M. Loree

**Affiliations:** 1 Medical Oncology Department, BC Cancer–Vancouver, Vancouver, Canada.; 2 Division of Medical Oncology and Hematology, Princess Margaret Cancer Centre, University Health Network, Toronto, Canada.; 3 Pathology Department, Vancouver General Hospital, Vancouver, Canada.; 4 Department of Hematology, BC Cancer–Vancouver, Vancouver, Canada.; 5 Medical Oncology Department, Providence Cancer Institute, Portland, Oregon.; 6 Medical Oncology Department, Ottawa Hospital Cancer Centre, Ottawa, Canada.; 7 Canadian Cancer Trials Group, Kingston, Canada.

## Abstract

Clonal hematopoiesis of indeterminate potential (CHIP) is the clonal expansion of hematopoietic stem cells from somatic mutations. It is a common incidental finding in cell-free DNA (cfDNA). We investigated the incidence of CHIP in cfDNA from patients with solid tumors and explored its association with treatment outcomes and adverse events. We reviewed cfDNA results from a local prospective solid tumor cohort (PREDiCT-l) and two randomized trials: Canadian Cancer Trials Group CO.26 [durvalumab + tremelimumab (D + T) or best supportive care in metastatic colorectal cancer] and Canadian Cancer Trials Group PA.7 (gemcitabine and nab-paclitaxel ± D + T in metastatic pancreatic adenocarcinoma). CHIP+ was defined as any mutation in *DNMT3A*, *TET2*, or *ASXL1* with a variant allele frequency ≥2%. Presumed germline variants (variant allele frequency >40%) were removed. The first line of treatment after cfDNA was reviewed for grade ≥3 and dose-limiting toxicities. The prevalence of CHIP in the 465 included patients was 10% to 30%, and it was more common as age increased (*P* = 0.003). *DNMT3A* was the gene most frequently mutated in all cohorts. Patients with CHIP in PA.7 treated with immunotherapy showed an improved progression-free survival versus CHIP− [HR = 0.55 (0.28–1.07); *P* = 0.079, *P*-interaction = 0.098 (multivariable)]. However, patients with CHIP treated with chemotherapy in PREDiCT-l showed a trend toward worse progression-free survival [HR = 1.82 (0.98–3.38); *P* = 0.059]. There was no difference in adverse event rates between CHIP ± groups for those treated with chemotherapy or immunotherapy. CHIP is common in patients with solid tumors. Although not appearing to affect rates of adverse events, CHIP may affect outcomes from immunotherapy or chemotherapy.

**Significance:**

Liquid biopsy is increasingly being used in oncology for tumor molecular characterization. CHIP is a common incidental finding in cfDNA, and its prevalence increases with age. This study builds on growing evidence of common CHIP variants in patients with solid tumors. The results suggest a possible clinical impact of CHIP on treatment outcomes from immunotherapy or chemotherapy. This may have implications for treatment selection for patients with solid tumors.

## Introduction

Clonal hematopoiesis of indeterminate potential (CHIP) is the acquisition of somatic mutations in hematopoietic stem cells that lead to clonal expansion, without unexplained cytopenia or an underlying hematologic malignancy (1–3). A single hematopoietic stem cell in a healthy person acquires approximately one protein-coding mutation per decade of life (4). CHIP is more common with aging as cells acquire more mutations and clones expand to reach the level of detection. In practice, CHIP is commonly identified as an incidental finding in cell-free DNA (cfDNA) from patients with solid tumors. DNA from hematopoietic stem cells containing CHIP variants enter the bloodstream and are mixed with other types of circulating DNA (such as circulating tumor DNA) in plasma. When a cfDNA analysis is undertaken, the CHIP variants create a biological “background noise” that can confound clinical interpretation.

Studies suggest that CHIP is detected in 5% to 40% of patients with solid tumors, and prevalence increases with age and prior cancer treatment (5–9). The most common CHIP mutations are those in myeloid cancer driver genes, including *DNMT3A*, *TET2*, and *ASXL1*, and confer loss of protein function. Importantly, mutations in DNA repair genes such as *TP53* and *ATM* can be misinterpreted as either tumor-derived (somatic) variants, CHIP variants, or germline variants because of their high alteration rate in solid tumors (∼37% of patients with *TP53* variants and ∼6% of patients with *ATM* variants seen in all solid tumors; refs. 5, 8, 10).

The presence of CHIP has been associated with an increased risk of atherosclerosis, thrombogenesis, cardiovascular disease, acute kidney injury, and the development of hematologic malignancies (1, 4, 11, 12). In addition, patients with hematologic malignancies who are CHIP+ have an increased risk of adverse events and nonrelapse mortality after autologous stem cell transplant or chimeric antigen receptor T-cell therapy (13–15). Little is known of the impact of CHIP on treatment outcomes and adverse events in patients with solid tumors. CHIP mutations can lead to increased expression of inflammatory genes in innate immune cells that create pro-inflammatory states (16). This resultant dysregulation of systemic inflammation provides a potential mechanism by which the presence of CHIP may affect outcomes or adverse events from cancer treatments.

The purpose of this study was to explore the incidence of CHIP in patients with solid tumors and investigate for an association among the presence of CHIP, cancer treatment outcomes, and adverse events.

## Materials and Methods

### Study design and patient cohorts

Patients in three study cohorts were included in this analysis:

1. Canadian Cancer Trials Group (CCTG) CO.26 (NCT02870920), a randomized trial of durvalumab + tremelimumab [immune checkpoint inhibitors (ICI)] or best supportive care (BSC) in patients with metastatic colorectal cancer, *n* = 168 (17);
2. CCTG PA.7 (NCT02879318), a randomized trial of gemcitabine and nab-paclitaxel (Chemo) with ICIs or Chemo alone in patients with metastatic pancreatic adenocarcinoma, *n* = 173 (18);
3. PREDiCT-l, a local real-world cohort of patients with advanced solid tumors, *n* = 124, who received physician’s choice of first-line systemic therapy for metastatic disease, which included Chemo, ICIs, ICIs + Chemo, or targeted therapy.

All patients included had cfDNA results available for review. Demographic data and baseline tumor characteristics were recorded. Progression-free survival (PFS), defined in CO.26 and PA.7 as the time from the point of randomization and in PREDiCT-l as the time from starting the first line of systemic therapy after cfDNA testing, until disease progression or death, was documented. Where available, overall survival (OS) was recorded, defined as the time from the point of randomization until death. For the CCTG CO.26 and PA.7 patients, grade ≥3 adverse events were included for analysis, and for the PREDiCT-l patients, dose-limiting adverse events were included. Data collection and assessment of PFS for patients in the PREDiCT-l cohort was performed using the hospital’s electronic health record. For patients in the two randomized trial cohorts, study treatment, PFS, and adverse event data were recorded from the study database.

Approval was granted by the local ethics board at BC Cancer for the use of the patient and sequencing data from the three included studies for this analysis. The analysis undertaken adhered to the guidelines from the Equator Network, using the TRIPOD checklist (Supplementary Table S1).

### Identification of CHIP variants

Given that each cohort had results from distinct cfDNA tests, and variants were annotated independently by respective companies, we implemented a set of rules across all cohorts to call CHIP variants. The sequencing data for each included patient was available and this was used as the source for identifying CHIP variants. The genes *DNMT3A*, *TET2*, and *ASXL1* were selected as they are the most common drivers of CHIP (all are epigenetic regulators predicted to have a similar functional impact and are infrequently mutated in solid tumors; ref. 5) and were included on all three panels. *TP53* and *ATM* were also considered but were excluded because of their high frequency of somatic mutation in the tumor types present in our cohorts (*TP53* ∼37% solid tumor types; *ATM* ∼6% solid tumors, ∼12% in colorectal; refs. 10, 19, 20).

Variants with ≥2% variant allele frequency (VAF) were considered to be CHIP. All CHIP variants are included in Supplementary Table S1. Indels in *ASXL1* at the homopolymer locus G646 were excluded as these have commonly been reported to be PCR artifacts and were common (>7%) across all three cohorts (19). Variants with ≥40% VAF were removed as potential germline alterations, and variants with a population frequency ≥0.001 were also excluded (21). Population frequency was queried from the gnomAD databases (version 4.0, whole-genome sequencing and whole-exome sequencing, RRID:SCR_014964) using gnomad-db v.0.1.4 (22, 23). PyLiftover (v0.4.1) was used to liftover hg19 to hg38 to query gnomAD.

Expanded CHIP variants were defined as those with a VAF ≥10%. High impact CHIP variants were defined as those predicted to disrupt the structure and/or functionality of the resulting protein, including frameshift and nonsense alterations.

### Statistical analysis

All statistical tests were performed in R version 3.6.3. Survival analyses were conducted using the survival (RRID:SCR_021137 v3.1.8) and survminer (RRID:SCR_021094 v0.4.7) packages. Comparisons of age were performed using Wilcoxon rank sum tests, and enrichment tests between CHIP ± patients, and adverse events used Fisher exact tests. *P* values were considered to be significant at <0.05. Interaction *P* values for Cox proportional hazard models were determined using multivariable models including age (±65 years), Eastern Cooperative Oncology Group (ECOG) status, sex, and tumor type (for PREDiCT-l). The significance threshold for interaction terms in the CCTG trials PA.7 and CO.26 was <0.1, which was acceptable because of the exploratory nature of the analysis aiming to identify potential signals for investigation in future studies, as this was the alpha set for the power calculations in both clinical trial designs.

### Data availability

The data used for this study were part of clinical trials (CO.27 and PA.7) and a prospective study (PREDiCT-l). An investigator who wishes to analyze data from this work must make a formal request to the CCTG (CO.27 and PA.7) and BC Cancer (PREDiCT-l). Requests will be reviewed according to the institutions’ Data Sharing and Access Policy.

## Results

### Patient characteristics

**Figure 1:**
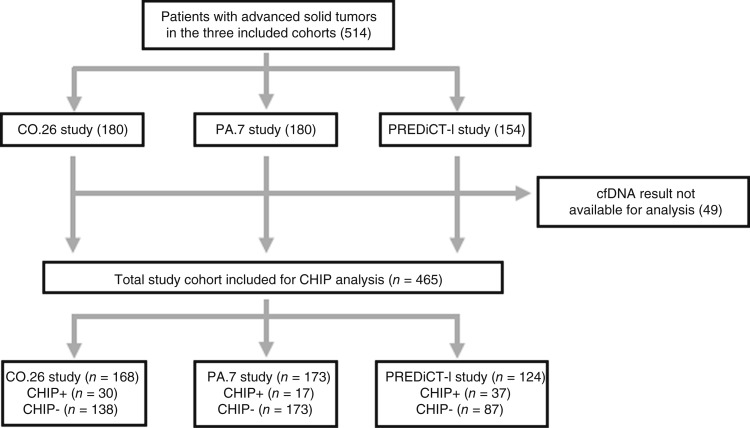
Consort diagram and reasons for exclusion.

A total of 514 patients participated in the three studies. Of these, 49 patients were excluded as there were cfDNA results available (see Fig. 1). The final CHIP analysis included 465 patients (*N* = 168, 173, and 124 patients from CO.26, PA.7, and PREDiCT-l, respectively).

The baseline characteristics and categories of treatments received are summarized in Table 1. There were fewer females than males in the CO.26 trial, but there were a larger proportion of females in the PREDiCT-l study (*P* < 0.001). The ECOG score was higher in the PREDiCT-l cohort compared with that in the two randomized trial cohorts (*P* < 0.001). There was no difference between the cohorts with regard to median age.

**Table 1:** Baseline characteristics of the included patients

Characteristic *n* (%^a^)	CO.26 (GuardantOMNI)			PA.7 (PredicineATLAS)			PREDiCT-l (FoundationOne Liquid CDx)		
	CHIP+	CHIP−	All	CHIP+	CHIP−	All	CHIP+	CHIP−	All
	*n* = 30	*n* = 138	*n* = 168	*n* = 17	*n* = 156	*n* = 173	*n* = 37	*n* = 87	*n* = 124
Female sex	10 (33)	46 (33)	56 (33)	8 (47)	77 (49)	85 (49)	29 (78)	57 (66)	86 (69)
Age ≥65 years	17 (57)	74 (54)	91 (54)	10 (59)	78 (50)	88 (51)	27 (73)	47 (54)	74 (60)
ECOG status:									
0	10 (33)	36 (26)	46 (27)	3 (18)	36 (23)	39 (23)	8 (22)	21 (24)	29 (23)
1	20 (67)	102 (74)	122 (73)	14 (78)	120 (77)	134 (77)	20 (54)	50 (57)	69 (56)
2	0	0	0	0	0	0	6 (16)	14 (16)	20 (16)
3	0	0	0	0	0	0	3 (8)	0	3 (2)
4	0	0	0	0	0	0	0	2 (23)	2 (2)
Tumor type:									
Colorectal	30 (100)	138 (100)	168 (100)	0	0	0	11 (30)	40 (46)	51 (41)
Pancreas	0	0	0	17 (100)	156 (100)	173 (100)	0	0	0
NSCLC	0	0	0	0	0	0	15 (41)	21 (24)	36 (29)
Ovarian	0	0	0	0	0	0	6 (16)	10 (11)	16 (13)
Other	0	0	0	0	0	0	5 (14)	16 (18)	21 (17)
Treatment:									
Chemo	0	0	0	4 (24)	54 (35)	58 (34)	22 (59)	58 (67)	80 (65)
ICI ± Chemo/TT	19 (63)	99 (72)	118 (70)	13 (76)	102 (65)	115 (66)	7 (19)	15 (17)	22 (18)
TT	0	0	0	0	0	0	4 (11)	11 (13)	15 (12)
BSC	11 (37)	39 (28)	50 (30)	0	0	0	0	0	0
No treatment	0	0	0	0	0	0	3 (8)	4	7 (6)

Abbreviation: TT, targeted therapy.

a All percentages are column percents.

### CHIP prevalence and associated factors

**Figure 2:**
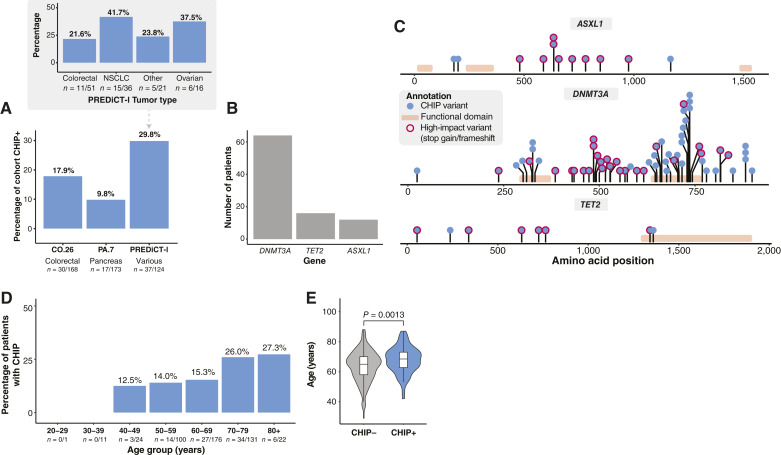
Prevalence of CHIP variants across cohorts and CHIP association with age. **A,** Percentage of patients with CHIP variants in each cohort. Insert shows the breakdown by tumor type in the PREDiCT-l cohort. **B,** Number of patients with a CHIP variant in each gene; *DNMT3A*, *TET2*, and *ASXL1*. **C,** Protein position of coding variants by gene. Each lollipop represents a patient, multiple dots show patients with the same variant position, and red outlines show variants of high impact (frameshift or stop gain). Orange bars along the length of the genes show functional domains. **D,** Percentage of patients with CHIP variants by age group. **E,** Age difference between patients with CHIP (CHIP+) and without CHIP (CHIP−). *P* value was determined by the Wilcoxon rank sum test.

Across the three cohorts, the prevalence of CHIP was 10% to 30% (Fig. 2A), with an average of 18% across all three (*N* = 85/465). The PREDiCT-l cohort had the highest prevalence of patients with CHIP, and this was most commonly seen in patients with non–small cell lung cancer (NSCLC) and ovarian cancer. Fifteen patients across all three cohorts (3.2%) had more than one CHIP variant, with one patient having three variants.

*DNMT3A* was the CHIP-associated gene most frequently mutated in all cohorts (Fig. 2B; Supplementary Table S2). The VAF of CHIP variants was similar across all panels [median 4.1% (PA.7), 4.22% (PREDiCT-l), and 4.56% (CO.26), range 2.03%–36.1%; *P* = 0.69; Supplementary Fig. S1]. Figure 2C shows the genomic positions of each of the identified CHIP variants. Within each gene, the variants occur across the length of the whole protein, both in functional and nonfunctional regions, with a few hotspot regions identified. *DNMT3A* had more variants in functional domains (48% vs. 25% *TET2*, 0% *ASXL1*). The proportion of CHIP variants that were clonally expanded (VAF ≥10%) was 23.3% in CO.26 (*N* = 7/30; *N* = 6 that received ICIs and *N* = 1 BSC), 23.53% in PA.7 (*N* = 4/17; *N* = 4 ICIs and *N* = 0 Chemo), and 18.92% in PREDiCT-l (*N* = 7/37; *N* = 3 ICIs and *N* = 4 Chemo). The proportion of variants that were considered to be high-impact protein-damaging CHIP variants (see “Materials and Methods”) was 50.0% in CO.26 (*N* = 15/30; *N* = 8 ICIs and *N* = 7 Chemo), 70.59% in PA.7 (*N* = 12/17; *N* = 9 ICIs and *N* = 3 Chemo), and 43.24% in PREDiCT-l (*N* = 16/37; *N* = 4 ICIs and *N* = 12 Chemo).

The prevalence of CHIP increased as age increased (Fig. 2D), with the highest rates seen in patients 80 years or older (27.3%). We did not observe CHIP variants in patients aged less than 40 years (*N* = 0/12 patients). The median age of patients with CHIP was significantly higher than that in patients without CHIP (65 vs. 68.5 years; *P* = 0.0013; Fig. 2E). There was no association between the presence of CHIP and sex (CO.26, *P* = 1; PA.7, *P* = 1; PREDiCT-l, *P* = 0.20) or ECOG status in any cohort (CO.26, *P* = 0.50; PA.7, *P* = 0.77; PREDiCT-l, *P* = 0.14).

### Impact on treatment outcomes

**Figure 3:**
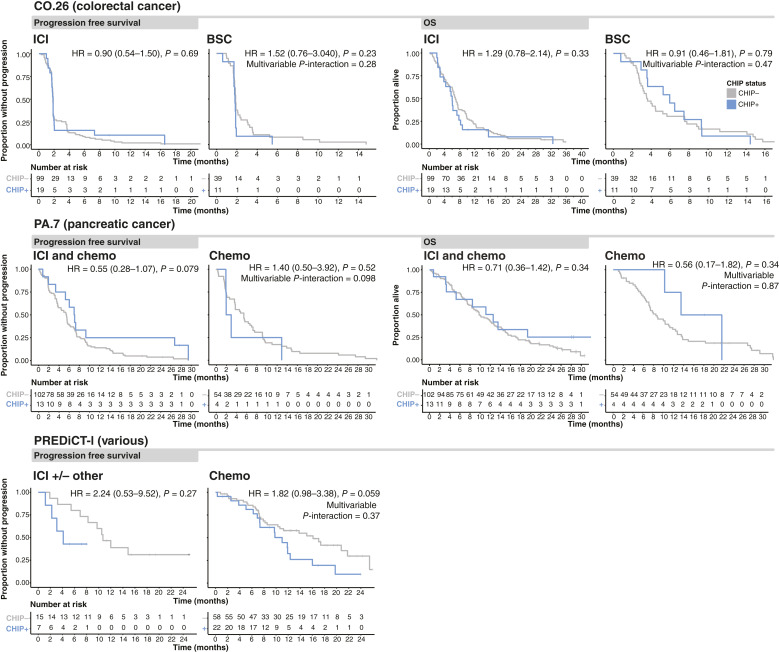
CHIP and treatment outcomes. Kaplan–Meier curves showing the difference in outcome between patients with (CHIP+) and without CHIP (CHIP−) and outcomes on different therapies. PFS is shown on the left two columns for all three cohorts, and OS is on the right two columns for CO.26 (top row) and PA.7 (middle row). Hazard ratios displayed are derived from univariable Cox proportional hazards models, and interaction *P* values are from multivariable models (see “Materials and Methods”).

All patients had data available for the PFS analysis. Patients with CHIP in PA.7 treated with ICIs showed improved PFS compared with patients without CHIP [HR = 0.55 (95% confidence interval = 0.28–1.07); *P* = 0.079, *P*-interaction = 0.098 (multivariable), see “Materials and Methods”; Fig. 3], and the same was not observed in Chemo-treated patients [HR = 1.40 (0.50–3.92); *P* = 0.52]. Conversely, patients with CHIP in PREDiCT-l treated with Chemo showed a nonsignificant worsening of PFS [HR = 1.82 (0.98–3.38); *P* = 0.059]. There was no association between CHIP status and PFS with ICI therapy in PREDiCT-l for the whole cohort [HR = 2.24 (0.53–9.52); *P* = 0.27]. However, all patients with CHIP receiving ICIs in this cohort had NSCLC, and when specifically examining the NSCLC subgroup, a worse PFS was observed (although not statistically significant) for those with CHIP versus without CHIP [HR = 2.25 (0.72–56.45); *P* = 0.096; Supplementary Fig. S2]. For patients in the CO.26 trial, there was no association between CHIP and PFS in either the ICI-treated arm [HR = 0.90 (0.54–1.50); *P* = 0.69] or the BSC arm [HR = 1.52 (0.76–3.040); *P* = 0.23, *P*-interaction = 0.28 (multivariable)].

OS was the primary endpoint of the CO.26 and PA.7 trials, and all patients in these trials had data available for the OS analysis. The presence of CHIP did not significantly affect OS for patients in either study cohort (Fig. 3). OS was not collected for the patients in the PREDiCT-l cohort.

Treatment outcomes for patients with high-impact CHIP variants or clonally expanded variants were not explored in the study because of the small sample sizes.

### Impact on treatment adverse events

**Figure 4:**
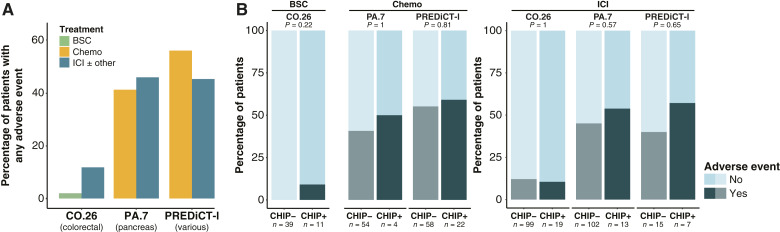
CHIP and adverse events. **A,** Percentage of patients on each treatment that had an adverse event on therapy, split by cohort and treatment. **B,** Proportions of patients with adverse events split by CHIP status. Lighter shades indicate no adverse event, and darker shades indicate an adverse event. *P* values were determined by Fisher exact tests.

The most common adverse events observed across the cohorts were rash, gastrointestinal toxicities, and bleeding or clotting abnormalities. The overall rates of adverse events were higher in the PA.7 and PREDiCT-l studies compared with CO.26 (Fig. 4A).

No significant difference was observed in the rates of adverse events between patients with CHIP versus without CHIP, either in those treated with BSC (CO.26), Chemo (PA.7 and PREDiCT-l), or ICIs (all cohorts, Fig. 4B).

## Discussion

This study examined the presence of CHIP (in *DNMT3A*, *ASXL1*, and *TET2*) in patients with solid tumors from three prospective clinical cohorts. CHIP variants were common, found in up to 30% of patients. The presence of CHIP may affect clinical outcomes in a treatment- and tumor type-specific manner, with patients with CHIP in PA.7 treated with ICIs showing an improved PFS compared with patients without CHIP, but a worse PFS observed in PREDiCT-l for patients with CHIP treated with Chemo, although not statistically significant. There was no association between CHIP and the development of adverse events from Chemo, BSC, or ICI therapy.

A more robust method for differentiating CHIP from tumor-derived variants in cfDNA is by performing concurrent white blood cell and plasma sequencing. One recent small study (*n* = 109) in patients with NSCLC used this approach and demonstrated that 25% of total variants detected in cfDNA were related to CHIP (24). Another recent study (*n* = 234) examined CHIP using genomic DNA isolated from peripheral blood mononuclear cells in patients with breast cancer (25). CHIP was identified in 15% of patients before treatment, and the emergence of CHIP on treatment was rare. There is ongoing research regarding how to accurately differentiate CHIP variants from tumor-derived variants in cfDNA from patients with solid tumors, without access to concurrent white blood cell sequencing. The most frequent CHIP-associated mutations occur in genes commonly altered in solid tumors, making the distinction between CHIP and somatic variants difficult without leukocyte-specific sequencing. Utilizing machine learning is one possible approach that may allow for accurate classification of CHIP variants from plasma cfDNA alone. In one study of over 4,000 patients with advanced cancer, a machine learning model identified CHIP variants in 30% of patients (26). As further data are gathered on CHIP variants, identification of CHIP based on specific variants may enable more accurate classification as likely CHIP versus somatic or germline (6).

Regarding clinical outcomes, a recent breast cancer study found that the presence of CHIP had no impact on survival in patients treated with Chemo (25). Interestingly, Chemo pressure was selective for the emergence of high-risk *TP53*-mutant clones with low variant allele fraction; however, the risk of developing treatment-related hematologic malignancies was low. The PREMIS trial (*n* = 127) in skin, genitourinary, and lung cancers utilized a 74-gene panel from whole blood. CHIP variants were detected in 43% of patients, and no association was found with PFS or OS with monotherapy ICI treatment (27). This differs slightly from our study’s findings and indicates that the association of CHIP with treatment outcomes may be linked to the specific ICI or Chemo received, tumor type, patient ECOG status, or other underlying genetic differences, warranting further exploration and more data generation (6).

This analysis represents a broad overview of CHIP prevalence in three distinct real-world cohorts and clinical trials and associated outcomes in patients with solid tumors. One strength of this study is the conservative approach to filtering, using population frequencies of variants, exclusion of variants likely to be germline or associated with known PCR artifacts, which increased the robustness of CHIP identification. Notably, although the PREMIS study found no association between CHIP and ICI survival outcomes, it did not use such strict filtering on defining CHIP status (27). The limitations of this study include the retrospective analysis with associated selection bias, the use of cfDNA instead of sequencing focused on the buffy coat, and restriction of the analysis to variants in three genes. Additionally, as this study utilized short-read sequencing, larger structural variants were not able to be confidently identified (such as those associated with lymphoid-related clonal hematopoiesis). Although this study is one of few exploring the role of CHIP in patients with solid tumors, one of the important limitations is the small sample size. This limits the analysis to being primarily descriptive. Although the three included cohorts were from prospective trials, CHIP analysis was not a prespecified objective of any of these studies. In addition, each study used a different cfDNA assay, which likely encompasses different in-house filtering of variants prior to the data being returned for this analysis. We expect that our stringent filtering steps, focus on the three genes most linked to CHIP, and analysis of each cohort separately limited the impact of this as witnessed by the similar CHIP frequencies and VAF levels.

### Conclusion

Overall, this study highlights that CHIP, an incidental finding from cfDNA assays, is found at varying frequencies in patients with solid tumors and may have tumor- and treatment-specific influence on outcomes.

## Supplementary Material

Supplemental Figure 1 VAF of all CHIP variants split by cohort

Supplemental Figure 2 Progression free survival for patients on PREDiCT-l, split by tumor type. Therapy received is indicated in each title above each Kaplan Meier curve.

Supplemental Table 1 TRIPOD checklist

Supplemental Table 2 All CHIP variants identified in the study
