# Supplementary figures and images for: Clonal Hematopoiesis of Indeterminate Potential and its Association with Treatment Outcomes and Adverse Events in Patients with Solid Tumors
Source: Cancer Res Commun. 2025 Jan 9;5(1):66–73. doi: 10.1158/2767-9764.CRC-24-0522 (PMC11713863; doi:10.1158/2767-9764.CRC-24-0522)

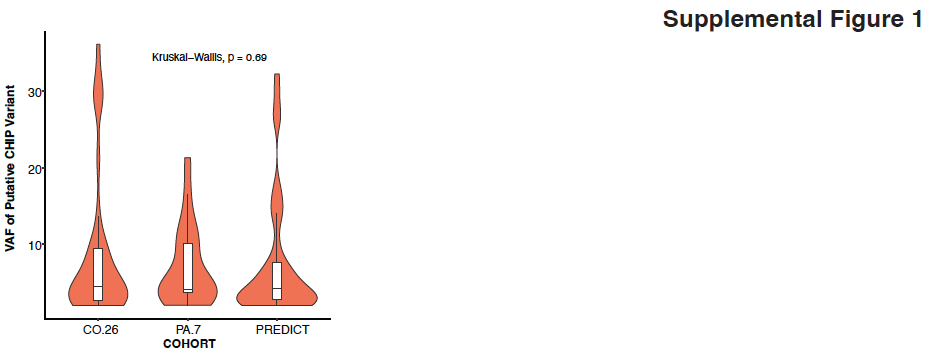

Supplement: Supplemental Figure 1 — VAF of all CHIP variants split by cohort [file crc-24-0522_supplemental_figure_1_suppsf1.png]

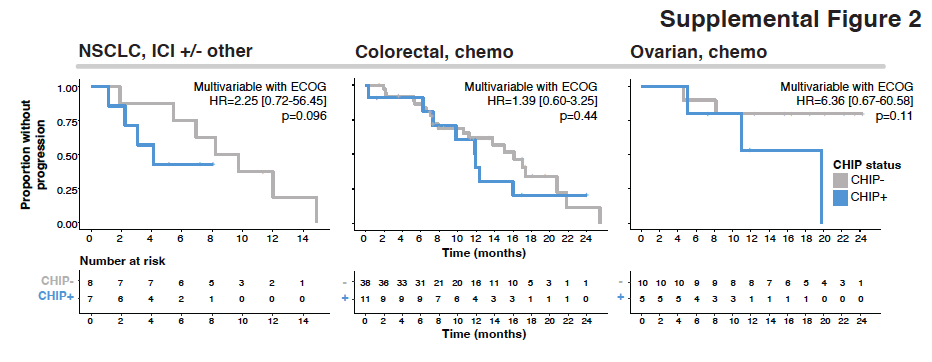

Supplement: Supplemental Figure 2 — Progression free survival for patients on PREDiCT-l, split by tumor type. Therapy received is indicated in each title above each Kaplan Meier curve. [file crc-24-0522_supplemental_figure_2_suppsf2.png]
